# Appearance of microvascular obstruction on high resolution first-pass perfusion, early and late gadolinium enhancement CMR in patients with acute myocardial infarction

**DOI:** 10.1186/1532-429X-11-33

**Published:** 2009-08-21

**Authors:** Adam N Mather, Timothy Lockie, Eike Nagel, Michael Marber, Divaka Perera, Simon Redwood, Aleksandra Radjenovic, Ansuman Saha, John P Greenwood, Sven Plein

**Affiliations:** 1 Division of Cardiovascular and Neuronal Remodelling, University of Leeds, UK; 2 Cardiovascular Division, The Rayne Institute, King's College London, St Thomas' Campus, London, UK; 3 Division of Imaging Sciences, The Rayne Institute, King's College London, St Thomas' Campus, UK; 4 Division of Medical Physics, University of Leeds, UK

## Abstract

**Background:**

The presence and extent of microvascular obstruction (MO) after acute myocardial infarction can be measured by first-pass gadolinium-enhanced perfusion cardiovascular magnetic resonance (CMR) or after gadolinium injection with early or late enhancement (EGE/LGE) imaging. The volume of MO measured by these three methods may differ because contrast agent diffusion into the MO reduces its apparent extent over time. Theoretically, first-pass perfusion CMR should be the most accurate method to measure MO, but this technique has been limited by lower spatial resolution than EGE and LGE as well as incomplete cardiac coverage. These limitations of perfusion CMR can be overcome using spatio-temporal undersampling methods. The purpose of this study was to compare the extent of MO by high resolution first-pass *k-t *SENSE accelerated perfusion, EGE and LGE.

**Methods:**

34 patients with acute ST elevation myocardial infarction, treated successfully with primary percutaneous coronary intervention (PPCI), underwent CMR within 72 hours of admission. *k-t *SENSE accelerated first-pass perfusion MR (7 fold acceleration, spatial resolution 1.5 mm × 1.5 mm × 10 mm, 8 slices acquired over 2 RR intervals, 0.1 mmol/kg Gd-DTPA), EGE (1-4 minutes after injection with a fixed TI of 440 ms) and LGE images (10-12 minutes after injection, TI determined by a Look-Locker scout) were acquired. MO volume was determined for each technique by manual planimetry and summation of discs methodology.

**Results:**

*k-t *SENSE first-pass perfusion detected more cases of MO than EGE and LGE (22 vs. 20 vs. 14, respectively). The extent of MO imaged by first-pass perfusion (median mass 4.7 g, IQR 6.7) was greater than by EGE (median mass 2.3 g, IQR 7.1, p = 0.002) and LGE (median mass 0.2 g, IQR 2.4, p = 0.0003). The correlation coefficient between MO mass measured by first-pass perfusion and EGE was 0.91 (p < 0.001).

**Conclusion:**

The extent of MO following acute myocardial infarction appears larger on high-resolution first-pass perfusion CMR than on EGE and LGE. Given the inevitable time delay between gadolinium administration and acquisition of either EGE or LGE images, high resolution first-pass perfusion imaging may be the most accurate method to quantify MO.

## Introduction

The basic aim of reperfusion therapy in acute myocardial infarction (AMI) is to restore normal blood flow to the ischemic myocardium as quickly as possible. Recanalisation of the infarct-related artery by primary percutaneous coronary intervention (PPCI), in particular, has seen significant reductions in mortality after AMI by limiting the amount of myocardial necrosis [1,2]. However, restoration of patency in the epicardial coronary artery sometimes fails to translate into recovery of myocardial function and in up to 30% of patients, reperfusion of the ischemic territory is incomplete due to myocardial microvascular obstruction (MO), which is seen angiographically as "no-reflow" [3]. Recent studies have demonstrated that MO plays a significant role in the pathophysiology of myocardial infarction and affects prognosis through an association with complications and poor recovery of left ventricular function [4-9].

Although there are several quantitative measures of angiographic perfusion such as corrected TIMI frame count [10], TIMI myocardial perfusion grade [11] and blush score [12], none of these directly visualise the affected myocardium. Gadolinium-enhanced CMR has emerged as an accurate tool to measure ventricular function, extent of infarction and MO [13,14]. The most common CMR technique to identify MO uses inversion recovery acquisition following intravenous administration of a gadolinium-based contrast agent. The preferred delay between gadolinium administration and image acquisition is 1-3 minutes ("early gadolinium enhancement", EGE), although many studies have measured MO from images acquired more than ten minutes after gadolinium administration ("late gadolinium enhancement", LGE). However, gadolinium chelates are freely permeable through the vascular wall and, therefore, will passively diffuse from normally perfused myocardium into the areas of microvascular damage over time. Hence, delayed imaging may underestimate the extent of MO.

Alternatively, MO can be identified by gadolinium enhanced first-pass myocardial perfusion CMR. In principle, first-pass perfusion would be expected to be the most accurate measure of MO size because it allows less time than either EGE or LGE for diffusion of gadolinium to artifactually diminish MO volume. Comparative studies of first-pass perfusion with LGE have confirmed this, showing larger MO extent by first-pass perfusion than LGE [15,16]. However, conventionally first-pass perfusion affords only about half the spatial resolution of EGE and LGE and typically does not cover the entire heart.

In this study, we sought to compare *k-t *SENSE accelerated first-pass perfusion with high spatial resolution and extended cardiac coverage with both EGE and LGE in the assessment of MO after acute myocardial infarction. We hypothesized that the appearance of MO on first-pass perfusion, acquired with the same spatial resolution as EGE and LGE and with comparable ventricular coverage, would be larger than with the other two methods.

## Methods

We recruited 34 consecutive patients hospitalised with first presentation acute ST elevation myocardial infarction and treated successfully with PPCI within 12 hours of symptom onset. Patients were recruited from two cardiac centres in the United Kingdom, Leeds General Infirmary and St. Thomas' Hospital, London. The study was approved by the institutional Research Ethics Committee and complied with the Declaration of Helsinki; written informed consent was obtained from all patients. Patients with a history of previous coronary revascularisation (i.e. PCI or coronary artery bypass surgery), previous myocardial infarction, renal failure or contraindication to CMR examination, were excluded.

### CMR protocol

All CMR studies were performed on Philips Achieva 1.5 Tesla MR systems (Philips Healthcare, Best, The Netherlands), using the same scanning protocol. All patients underwent CMR within 72 hours of reperfusion therapy. Cine images of the entire left ventricle were acquired using an ECG-gated balanced steady state free precession (SSFP) pulse sequence. Following this, an intravenous bolus dose of 0.1 mmol/kg Gd-DTPA (Dimeglumine gadopentetate, Magnevist, Bayer Schering Health Care Limited, UK) was administered at a rate of 5 ml/s by a power injector (Medrad Spectris Solaris, Medrad, USA). *k-t *SENSE accelerated first-pass perfusion imaging was performed simultaneously with the injection of gadolinium, using the following imaging parameters: Fast gradient echo, repetition time (TR) 3.7 ms, echo time (TE) 1.0 ms, flip angle 15°, 7-fold k-t acceleration, 11 training profiles, spatial resolution 1.5 mm × 1.5 mm × 10 mm, field of view (FOV) range 350-400 mm, 8 slices acquired in the left ventricular (LV) short axis over 2 RR intervals and no interslice gap.

Immediately after first-pass perfusion imaging, a second bolus dose of 0.1 mmol/kg Gd-DTPA was administered. EGE images were acquired 1-4 minutes after gadolinium injection with a fixed inversion time (TI) of 440 ms (inversion recovery-prepared T1-weighted gradient echo, TR 4.9 ms, TE 1.9 ms, flip angle 15°, spatial resolution 1.35 mm × 1.35 mm × 10 mm, FOV range 350-400 mm, 10 two-dimensional slices in LV short axis and no interslice gap). In our practice we routinely use a fixed TI of 440 ms as it provides excellent contrast between microvascular obstruction and normal myocardium. Normal myocardium appears bright compared with the low signal intensity of MO.

10 minutes after gadolinium injection, a 'Look Locker' sequence was performed to obtain the most appropriate TI to null the signal intensity of normal myocardium. The minimum TI for the study group was 225 ms and the maximum was 250 ms. The median TI was 240 ms. LGE images were then acquired 10-15 minutes after gadolinium injection with identical pulse sequence parameters as for EGE apart from the specifically determined TI.

### CMR analysis

The CMR images were analysed off-line using commercial software (MASS 6.0, Medis, Leiden, The Netherlands) by an observer blinded to all other data and all clinical details. For assessment of left ventricular function and mass, the end-diastolic and end-systolic cine frames were identified for each slice and the endocardial and epicardial borders were manually traced. The end-diastolic and end-systolic volumes were then calculated using Simpson's rule (i.e. sum of cavity sizes across all continuous slices).

For first-pass perfusion data, the frame demonstrating peak signal intensity in the remote normal myocardium was chosen for quantitation of the myocardial extent of the perfusion defect. The perfusion defect was defined visually as a hypoenhanced region and then planimetered manually. A total volume of MO was then calculated by Simpson's rule. The presence of dark rim artifacts is much reduced with high resolution first-pass perfusion sequences [17]. Where artifacts were present and occurred in the territory of the infarct, contours were drawn to exclude the artifacts. Papillary muscle was excluded from all analyses. Image quality of the first-pass perfusion images was assessed by consensus opinion (ANM and AS) and scored on a scale of 1 to 4, as follows: (1) unable to interpret results, (2) poor, (3) satisfactory and (4) very good.

**Figure 1:**
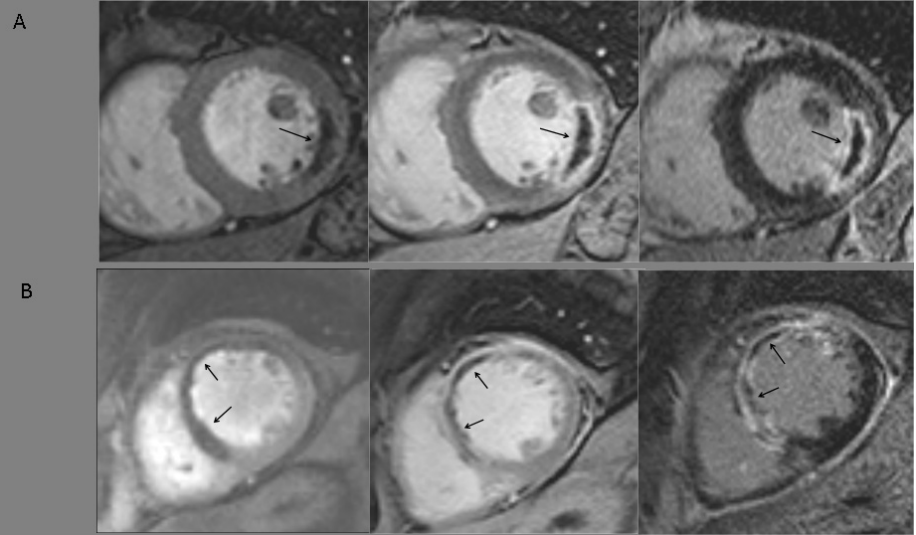
**A) CMR images from a patient with acute lateral myocardial infarction**. Arrows point to MO (areas of hypoenhancement) on first-pass perfusion (left), EGE (middle) and LGE (right). B) CMR images from a patient with acute anterior myocardial infarction. Arrows point to MO (areas of hypoenhancement) on first-pass perfusion (left), EGE (middle) and LGE (right).

EGE images were assessed in a similar manner. For each slice, the region of hypoenhancement, compared to normal myocardium, was defined visually and then planimetered manually. LGE images were assessed both for infarct size and MO. The endocardial and epicardial borders on each slice were traced manually. Infarcted tissue was defined as areas with late gadolinium enhancement. These regions were identified and then quantified using a semi-automated algorithm. Areas of enhancement were defined as myocardium with a signal intensity > 2 SD above the mean signal intensity of the remote normal myocardium [18]. The mass of infarcted myocardium was then automatically calculated. MO on LGE imaging was defined as a region of subendocardial hypoenhancement within the enhanced region (Figure 1). The area of MO was manually planimetered and a volume was calculated using Simpson's rule. All volumes were converted to mass by multiplying by the myocardial density (1.05 g/ml). For each method of assessment of MO, the transmural and circumferential extent of MO was measured in the slice that demonstrated the maximum area of MO.

In all patients, data acquisition was commenced at the same time points after gadolinium administration (i.e. at 1 minute for EGE and at 10 minutes for LGE). However, because EGE and LGE data are 2D stacks acquired over several breath holds, the acquisition duration of each stack varies with patients' breath-holding capacity and heart rate. Because the signal characteristics change over time in both EGE and LGE, we investigated whether the duration of the data acquisition could be a confounding variable for the results. We sought to determine if the greatest differences between first-pass perfusion and EGE and LGE measurements of MO were attributable to the cases which had the greatest image acquisition durations.

### Statistical analysis

Statistical analysis was performed using commercially available software (SPSS, version 15.0, SPSS Inc, Chicago, USA). Two-sided p values ≤ 0.05 were considered to be statistically significant. Data were compared using Student's paired t tests, independent samples t tests and Pearson's correlation coefficients. Continuous data are represented as median values and interquartile ranges (IQR). The agreements between the methods first-pass perfusion, EGE and LGE were demonstrated using Bland-Altman plots.

The reproducibility of MO assessment for each technique was assessed using a random sample of ten patients. In order to assess intra-observer variability, MO contours were redrawn several months after the original contours, and without reference to them, by the original observer (ANM). An additional observer (AS) independently drew contours using the same images in order to assess inter-observer variability. It was firstly confirmed that the degree of error between repeated measurements was not proportional to the magnitude of the measurements, using Kendall's correlation coefficient and scatter plots. The variance between repeated measurements of MO by first-pass perfusion, EGE and LGE was then calculated for each patient. The within-subject variance for each technique was obtained by taking the mean of the ten patients' variances. The within-subject standard deviation was then derived by performing the square root of the within-subject variance.

The intra-observer and inter-observer variabilities were also assessed by calculating the Coefficient of Variation (CV) or error within a single measurement estimated from repeated measurements. By using the formula: S_w _= S_d_/v2, where S_w _is the common within subject standard deviation and S_d _is the standard deviation of the differences between two measurements, CV was then calculated using the formula: CV = (S_w_/*x*) × 100, where *x *is the total mean of all measurements.

## Results

The baseline characteristics of the study population are summarised in Table 1. The median image quality score for the first-pass perfusion images was 3 (i.e. satisfactory). Only four cases were deemed to be of poor quality and all images were interpretable. The intra-observer variability and inter-observer variability assessments for the measurement of MO by the three different techniques are outlined in Table 2. Evidence of myocardial infarction by LGE was present in all cases. The median infarct mass was 21.9 g (IQR 11.4). The median left ventricular ejection fraction (LVEF) was 41.6% (IQR 7.7).

**Table 1:** Summary of baseline characteristics of study patients (n = 34)

N = 34	
Age (median, interquartile range)	63 (IQR 56-66)
Male %	2
Current smoking %	50
Family history of premature coronary artery disease %	29
Hypertension %	29
Hypercholesterolaemia %	35
Diabetes %	9
Infarct-related artery %	
Left anterior descending artery	32
Left circumflex artery	24
Right coronary artery	44
Primary percutaneous coronary intervention (PPCI) %	100
TIMI flow post-PCI %	
TIMI 3	85
TIMI 2	15
TIMI1	0
Use of Glycoprotein 2b/3a inhibitors %	53
Use of Bivalirudin %	47
Use of percutaneous thrombectomy device %	21

**Table 2:** Summary of the intra-observer variability and inter-observer variability assessments for the measurement of MO by first-pass perfusion, EGE and LGE.

**Method of MO assessment**	**Intra-observer variability**		**Inter-observer variability**	
	**Within-subject SD (g)**	**Coefficient of variation (%)**	**Within-subject SD (g)**	**Coefficient of variation (%)**
First-pass perfusion	0.65	5.2	0.54	6.54
EGE	0.48	8.14	0.29	4.89
LGE	0.2	6.29	0.32	10.16

**Figure 2:**
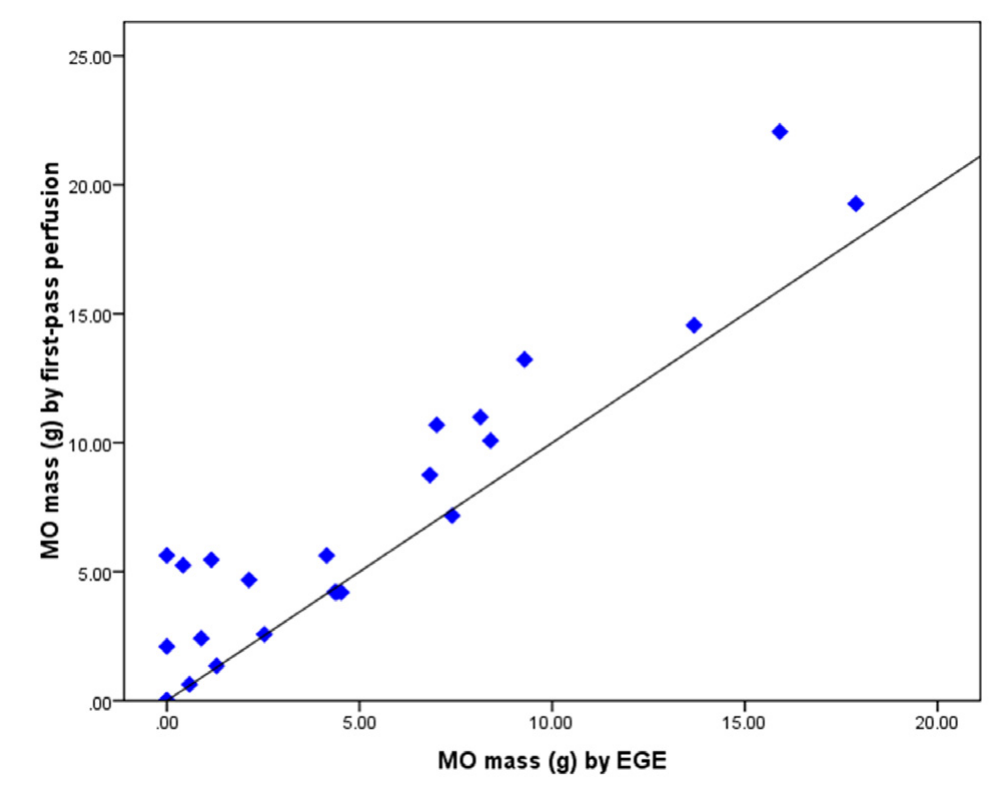
**Graph demonstrating correlation between MO mass (g) measured by k-t SENSE first-pass perfusion and EGE (r = 0.91)**. Diagonal black line represents the line of identity.

**Figure 3:**
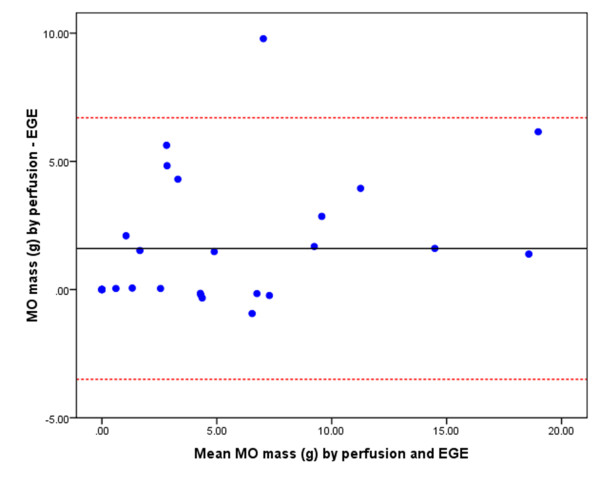
**Bland-Altman plot demonstrating the agreement of MO mass as measured by first-pass perfusion and EGE**. The central horizontal line represents the mean bias and the red dashed lines represent the limits of agreement (i.e. 2 SD from the mean difference of MO mass measured by first-pass perfusion minus EGE).

**Figure 4:**
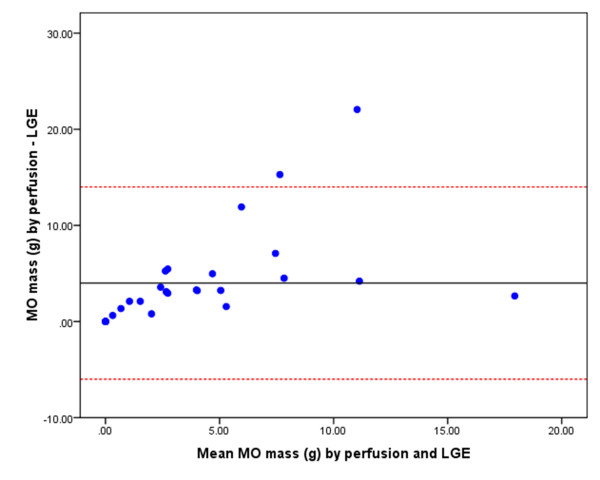
**Bland-Altman plot demonstrating the agreement of MO mass as measured by first-pass perfusion and LGE**. The central horizontal line represents the mean bias and the red dashed lines represent the limits of agreement (i.e. 2 SD from the mean difference of MO mass measured by first-pass perfusion minus LGE).

**Figure 5:**
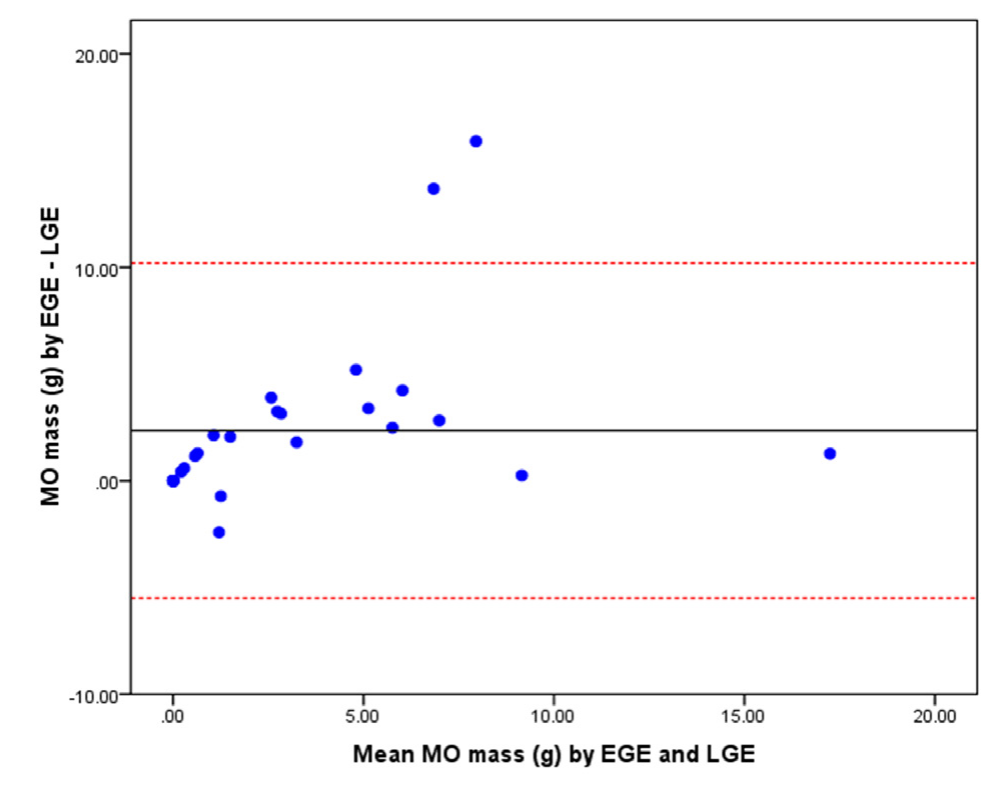
**Bland-Altman plot demonstrating the agreement of MO mass as measured by EGE and LGE**. The central horizontal line represents the mean bias and the red dashed lines represent the limits of agreement (i.e. 2 SD from the mean difference of MO measured by EGE minus LGE).

*k-t *SENSE first-pass perfusion detected more cases of MO than EGE and LGE (22 vs. 20 vs. 14, respectively). There were no cases where MO was detected by EGE or LGE and not by first-pass perfusion. The LV mass was consistent when measured by the three different imaging techniques. Therefore, the extent of MO is represented by the absolute measurement in grams. The extent of MO imaged by first-pass perfusion (median mass 4.7 g, IQR 6.7) was significantly greater than EGE (median mass 2.3 g, IQR 7.1, p = 0.002) and LGE (median mass 0.2 g, IQR 2.4, p = 0.0003). The extent of MO measured by EGE was greater than by LGE (p = 0.004). There was a positive correlation between MO mass measured by first-pass perfusion and EGE (r = 0.91, p < 0.001) (Figure 2) and between first-pass perfusion and LGE (r = 0.55, p = 0.002). The bias and limits of agreement between these methods of assessing MO are demonstrated in the Bland-Altman plots in Figure 3, Figure 4 and Figure 5.

The transmural extent of MO was greater in every case by first-pass perfusion, except in two patients where the greatest extent was measured by EGE. The circumferential extent of MO was largest by first-pass perfusion in all cases.

The minimum time to the first CMR scan was 28 hours and the maximum time was 72 hours (median 52 hours, IQR 26.25). There were no significant correlations between the time to first CMR scan and the MO/infarct size ratio measured by the three different CMR techniques (first-pass perfusion r = 0.37, p = 0.09; EGE r = 0.15, p = 0.52; LGE r = 0.26, p = 0.24).

We tested whether the image acquisition durations for EGE and LGE were related to the differences in the extent of MO measured by these techniques. The difference between MO mass as measured by first-pass perfusion and EGE did not correlate with the acquisition duration for EGE (r = 0.014, p = 0.95). Similarly, the difference between MO mass as measured by first-pass perfusion and LGE did not correlate with the acquisition duration for LGE (r = -0.39, p = 0.07). There was also no evidence of an exponential or logarithmic correlation.

In this study, 18 (53%) patients received a Glycoprotein (GP) IIb/IIIa inhibitor and 16 (47%) patients received Bivalirudin (direct thrombin inhibitor) as adjunctive pharmacotherapy during the PPCI procedure. There were no significant differences between these two treatment groups regarding the mass of MO measured by first-pass perfusion (median MO mass (g) (IQR), GP IIb/IIIa 5.63 (9.3) vs. Bivalirudin 2.42 (6.67), p = 0.12); EGE (GP IIb/IIIa 6.2 (10.2) vs. Bivalirudin 2.70 (6.82), p = 0.07 or LGE (GP IIb/IIIa 2.41 (2.38) vs. Bivalirudin 1.58 (3.43), p = 0.55).

## Discussion

This study shows that in patients with recent AMI, successfully treated with PPCI, high-resolution first-pass perfusion CMR identifies more cases of MO than EGE and LGE and that the extent of MO appears larger on first-pass perfusion than either EGE or LGE.

Angiographic assessments of MO such as corrected TIMI frame count and myocardial blush grade are well validated [19] but lack sensitivity and specificity. In addition, such methods do not allow direct visualisation of the affected myocardium and the extent of MO may continue to evolve over the hours and days following AMI meaning an assessment made immediately after reperfusion may lack accuracy [20]. The exposure of patients to ionising radiation and risk of procedural complications means that these methods are not suitable for serial monitoring which is needed to further our understanding of the pathophysiology as well as evaluate new treatments. In this regard, a non-invasive and safe imaging modality, such as CMR, is ideal.

CMR is the gold standard assessment of left ventricular mass and function [21] and has the best interstudy reproducibility of any technique for these assessments [22]. Gadolinium-enhanced CMR has also been used extensively in the study of myocardial infarction and MO [13,14,23,24]. Researchers have used different methods for detecting MO. Some studies have highlighted the prognostic significance of MO, defined by EGE or LGE, in predicting left ventricular remodeling, adverse cardiovascular complications and persistent functional impairment [6,25-27]. First-pass perfusion imaging has been used by others to demonstrate MO as a predictor of poor functional recovery after myocardial infarction [28,29].

There are two comparisons in the published literature between first-pass perfusion and LGE in the detection of MO. Both found that first-pass perfusion detected MO more frequently and to a greater extent [15,16]. Our research confirms these findings. However, both of these studies used LGE as the delayed enhancement technique rather than EGE. A further limitation of both studies was the incomplete left ventricular coverage by first-pass perfusion. Lund et al., used single slice perfusion and Yan et al., used 3 slices. Therefore, localised microvascular dysfunction was likely to be missed and this possibly accounted for small numbers of patients in both studies with evidence of MO by LGE but not first-pass perfusion.

In the present study, we compared high resolution accelerated *k-t *SENSE first-pass perfusion with both EGE and LGE methods. The accelerated perfusion sequence allowed almost full ventricular coverage in a single breath-hold by acquiring data over 2 RR intervals and the acquired spatial resolution was comparable to EGE and LGE, therefore overcoming the limitations of previous studies of first-pass perfusion. We suggest that high spatial resolution and good ventricular coverage were the reasons why we found more patients with MO on first-pass perfusion than EGE or LGE and demonstrated a significant difference in the extent of MO. In doing so, our observations support previous suggestions that gadolinium passively diffuses into areas of microvascular damage immediately following gadolinium injection and, therefore, the apparent size of MO may decrease over a relatively short time period after injection of a gadolinium-based contrast agent.

In the absence of a reference standard for *in vivo *assessment of MO, the differences we have observed in the apparent MO dimensions with different CMR methods may in part reflect technical differences between the three CMR methods. In addition, several physiological phenomena may affect the three CMR methods differently. On first-pass perfusion for example, old myocardial infarction may produce hypoenhancement, while it would lead to enhancement on EGE and LGE. In our sample, this is unlikely to have been a confounding factor as none of the study subjects had suffered previous MI. Rarely, at rest, first-pass perfusion can produce an area of hypoenhancement in a myocardial territory dependent on a tight coronary artery stenosis. In this study, however, there were no cases with perfusion defects in more than one coronary artery territory and in all cases the perfusion defects matched the territory of the infarct related artery. Finally, areas of an acute myocardial infarction may be dependent on collateral flow. It is possible that collateral flow dependent myocardium will demonstrate late gadolinium enhancement relative to the remote normal myocardium and that this could therefore, contribute to the greater extent of MO measured by first pass perfusion.

Rochitte et al.[20], demonstrated in dogs that the MO/infarct size ratio is not stable and increases over the first 48 hours after AMI. In order to minimize the potential influence of this phenomenon on comparisons made in our study, we aimed to perform CMR between 48 and 72 hours after presentation with AMI. Our results demonstrated that there were no significant correlations between MO/infarct size ratio and time to CMR scan for all three CMR techniques.

In this study, 29 (85%) patients had TIMI 3 flow and 5 (15%) patients had TIMI 2 flow following primary PCI. These findings are consistent with other studies, e.g. Nijveldt et al. (2008) [27] examined 60 patients with AMI treated successfully with PPCI, and found TIMI 3 flow in 82% and TIMI 2 flow in 18% of cases. They detected MO in 68% of cases. Therefore, microvascular dysfunction is not solely dependent on restoration of epicardial coronary artery patency. Ischemia itself causes ultrastructural damage to the microvasculature and electron microscopy has revealed endothelial abnormalities, neutrophil occlusion and marked erythrocyte stasis at the level of capillaries [3]. Indeed, successful reperfusion itself can lead to additional injury to the microvasculature by causing further neutrophil infiltration, free radical generation and activation of the complement system [9]. In addition, compression of the microvasculature by edematous and necrotic myocytes can increase distal vascular resistance and cause arteriolar spasm. Platelet microembolization following coronary artery plaque rupture and then PCI may also play a significant role [9].

Although there were no statistically significant differences between patients treated with GP IIb/IIIa inhibitors and Bivalirudin, the former treatment was associated with a trend to larger MO as assessed by all three CMR methods. These initial observations may warrant evaluation in larger future studies.

## Limitations

The present study did not compare the CMR methods of assessment of MO with a validated reference standard. However, this has been studied before with demonstration of good correlation between CMR and TIMI frame count [16]. Also, we did not match our findings to clinical outcome measures, although MO measured by first-pass perfusion and delayed enhancement CMR has been shown to correlate with infarct mass and left ventricular ejection fraction [16], which are both established prognostic markers following AMI.

## Conclusion

In conclusion, we demonstrated that high resolution first-pass perfusion CMR detected MO more frequently and to a greater extent than either early or late gadolinium enhancement CMR methods. We postulate that first-pass perfusion imaging is likely to give the most accurate assessment of MO, as it is not confounded by the diffusion of gadolinium into the no-reflow zone over time, which will lead to an underestimation of MO by any delayed acquisition, regardless of timing. By using *k-t *SENSE acceleration and acquisition over 2 RR intervals we were able to assess MO with the same spatial resolution and cardiac coverage as current EGE and LGE techniques, making first-pass perfusion a viable and more time-efficient alternative to these methods. Given that outcome after AMI is related to the presence and extent of MO, future studies are warranted to determine if the detection of MO by first-pass perfusion has added prognostic value over MO detected by EGE or LGE.

## Competing interests

The authors declare that they have no competing interests.

## Authors' contributions

ANM participated in the design of the study, performed the data collection and analysis and drafted the manuscript. TL collected data and revised the manuscript. AS analysed the data and revised the manuscript. EN, MM, DP, SR and JPG were all involved in revising the manuscript and giving final approval. AR was involved in designing the perfusion CMR pulse sequence used in this study. SP was responsible for the conception of the study and drafting the manuscript.
